# THE IMPACT OF CHANGES IN COGNITIVE FUNCTIONING ON SOCIAL ISOLATION

**DOI:** 10.1093/geroni/igad104.1670

**Published:** 2023-12-21

**Authors:** Jay Kayser, Lydia Li

**Affiliations:** University of Michigan, Ann Arbor, Michigan, United States; University of Michigan, Ann Arbor, Michigan, United States

## Abstract

This study examines if transitioning from normal to impaired cognitive functioning is associated with changes in social isolation among community-dwelling older adults without cognitive impairment at baseline. This study uses nine waves (2011-2019) of data from the National Health and Aging Trends Study (NHATS). The sample (N=8,245) was representative of Medicare beneficiary at baseline. Social isolation was measured using a seven-item index that captures a range of social activities and network characteristics. Cognitive status was assessed through self-report of having Alzheimer’s disease, the Dementia Screening Interview (AD8), and cognitive testing. Based on pre-determined criteria, participants were grouped as either cognitively intact or impaired. Fixed effect linear modeling with robust standard errors was used to examine the impact of transitioning from intact to impaired cognitive functioning on social isolation. At baseline 5,575 Participants were cognitively intact. At each successive wave, between 12% and 16% of the sample was cognitively impaired. After controlling for time-varying covariates, transitioning from intact to cognitive impairment was associated with a statistically significant increase in social isolation (β=.18, SE=.03, p <.001). Gender and educational attainment did not impact this association. These findings underscore the need for policies and interventions to increase social connection among older adults, particularly during early stages of cognitive impairment.

